# A randomised controlled trial comparing the outcomes of arteriovenous fistula for dialysis created under local vs regional anaesthesia: reflections on recruitment and process evaluation study

**DOI:** 10.1186/s13063-025-08945-0

**Published:** 2025-08-04

**Authors:** Emma Aitken, Cecilia Vindrola-Padros, Nicolas Hall, the ACCess study collaborators

**Affiliations:** 1 Department of Renal Surgery, Elizabeth University Hospital, 1345 Govan Road, Glasgow, Queen G51 4TF UK; 2 Rapid Research Evaluation and Appraisal Lab, University College London, London, UK

## Abstract

**Background:**

Despite the crucial role that randomised controlled trials (RCTs) play in establishing the efficacy of new treatments, many are terminated early due to difficulty with subject recruitment. Trials of complex perioperative interventions are notoriously difficult to deliver. We describe our experiences of successful recruitment into an RCT of anaesthetic technique in vascular access surgery with the aim of exploring barriers and enablers to efficient recruitment and trial delivery.

**Methods:**

A mixed-methods approach was adopted to evaluate the recruitment and implementation processes of The Anaesthesia Choice for Creation of artEriovenouS fiStulae (ACCess) study (https://doi.org/10.1136/bmjopen-2021-052188) (Deloitte. Patient Recruitment is Often the Holy Grail for Clinical Research…Could Virtual Trials Improve our Chances of Reaching It? 2020). Recruitment figures demonstrating trial progression and site set-up are reported quantitatively. Contemporaneous data was collected on site-specific challenges, particularly in relation to the COVID-19 pandemic. The Scottish Vascular Access Appraisal Service Evaluation Tool was utilised to summarise the pre-existing vascular access infrastructure at each site and inferences made about service “resilience”. An embedded process evaluation study, supplemented by the researchers own reflections, qualitatively explored motivators and challenges in trial set-up, recruitment and delivery utilising thematic analysis of semi-structured interviews with patients and health care practitioners (HCPs). A rapid feedback evaluation approach permitted within trial feedback to the main trial team.

**Results:**

Five hundred seventy-one patients with stage V CKD or on haemodialysis were successfully recruited from 20 UK-centres over a 2-year period, making this the largest RCT of vascular access in Europe to date. A “desire to improve care for patients with kidney disease” was the main motivator for participation amongst both patients and HCPs. Good communication, strong leadership, and simple trial documentation were viewed as important enablers; whilst staffing shortages and reduced access to theatre were considered the principal barriers. Sites that reported well-established MDT-working appeared best-able to mitigate against these difficulties. A pragmatic trial protocol, which could easily be implemented within existing clinical practice, was considered essential for many centres. Anxiety and uncertainty associated with dialysis initiation was evident in reasons for non-participation with 32% declining participation due to an unwillingness for randomisation. Furthermore, the workload associated with travel to study visits and completion of “tiresome” HR-QOL questionnaires were considered additional barriers in a patient group with significant pre-existing treatment burden.

**Conclusions:**

Despite anticipated challenges, target recruitment was achieved on schedule with predicted timelines. In depth knowledge of the patient population; early engagement of a broad multidisciplinary team; and a pragmatic protocol that could be effectively incorporated into routine clinical care proved fundamental to success. Consideration of these factors may be of benefit to other researchers designing clinical trials, especially within the renal population.

**Trial registration:**

Both the ACCess study and process evaluation study are registered with the respective clinical trials databases: ISRCTN14153938 and MRC SWAT Repository 150.

## Background

Randomised controlled trials (RCT) are considered to provide “gold standard” evidence, playing a crucial role in establishing the efficacy of new treatments. However, many are abandoned due to difficulty with subject recruitment. Around half of clinical trials are delayed [1] and at least one in three RCTs are discontinued [2], with poor recruitment cited as the leading cause for early termination [3, 4]. A recent systematic review by our own group concluded that clinical trials (particularly multicentre RCTs) of vascular access of haemodialysis endured a similar fate [5]. Incomplete, underpowered or delayed trials incur significant cost and may result in type 2 error rendering conclusions invalid. It is therefore essential that researchers share experiences of enablers and barriers to recruitment, in order to inform more efficient future trial design and to avoid ‘research waste’ [6].

The Anaesthesia Choice for Creation of arteriovEnouS fiStulae (ACCess) study is an NIHR-funded multicentre single-blinded randomised controlled trial (RCT) comparing the outcome of arteriovenous fistulae (AVF) for haemodialysis (HD) created under regional (RA) vs local (LA) anaesthetic [7]. It is the largest RCT of vascular access in Europe to date. The study aimed to recruit 566 patients from 20 centres across the UK over a 2-year period. Recruitment commenced in May 2021 and completed, on schedule, in May 2023.

The timing and context of the ACCess study provided some unique dilemmas for recruitment and trial delivery. Firstly, it is a study of a perioperative intervention delivered within an operating theatre environment that remained affected by the recent pandemic. In 2021–2022, over 30,000 operations in NHS England did not proceed due to staffing shortages [8] and the post-COVID-19 recovery plan for elective operative care was not published until May 2022 [9]. Secondly, the multidisciplinary nature of the research team is remarkable: anaesthetists, nephrologists, vascular access surgeons, sonographers and vascular access nurses are all involved. Atypically, the speciality providing the intervention (in this case anaesthetics) is not the one primarily concerned with the outcome (nephrology/vascular access surgery). Moreover, most study participants have chronic kidney disease stage 5 (CKD-V) and are within 6 months of needing to start HD. Like many health-state transitions, the “peri-dialysis start” period is a time of great anxiety and uncertainty [10, 11]. Furthermore, the need for regular dialysis sessions confers a significant workload and treatment burden [12, 13]. Capacity for ancillary activities, such as participation in clinical research, is likely limited [14].

Despite these challenges, the ACCess study has recruited to target and on schedule. Within this manuscript we will reflect on that relative success; and utilise an embedded qualitative study to consider barriers, enablers and motivators (for both patients and researchers) to recruitment within this patient population. We believe our experiences can valuably inform future researchers, within the fields of nephrology and perioperative care, to design and deliver more effective and efficient clinical trials.

## Methods

We present a mixed-methods approach. Our aims are threefold:

1. To describe the successful recruitment into the ACCess study in a quantitative manner.
2. To utilise qualitative data obtained from an embedded process evaluation study (supplemented by personal observations) to describe aspects of trial design, delivery and decision-making that may have helped facilitate recruitment.
3. To explore perceived barriers and enablers to recruitment across different sites within the ACCess study, highlighting areas that future researchers may wish to consider in trial design and delivery.

### Summary of the RCT (ACCess study)

**Fig. 1:**
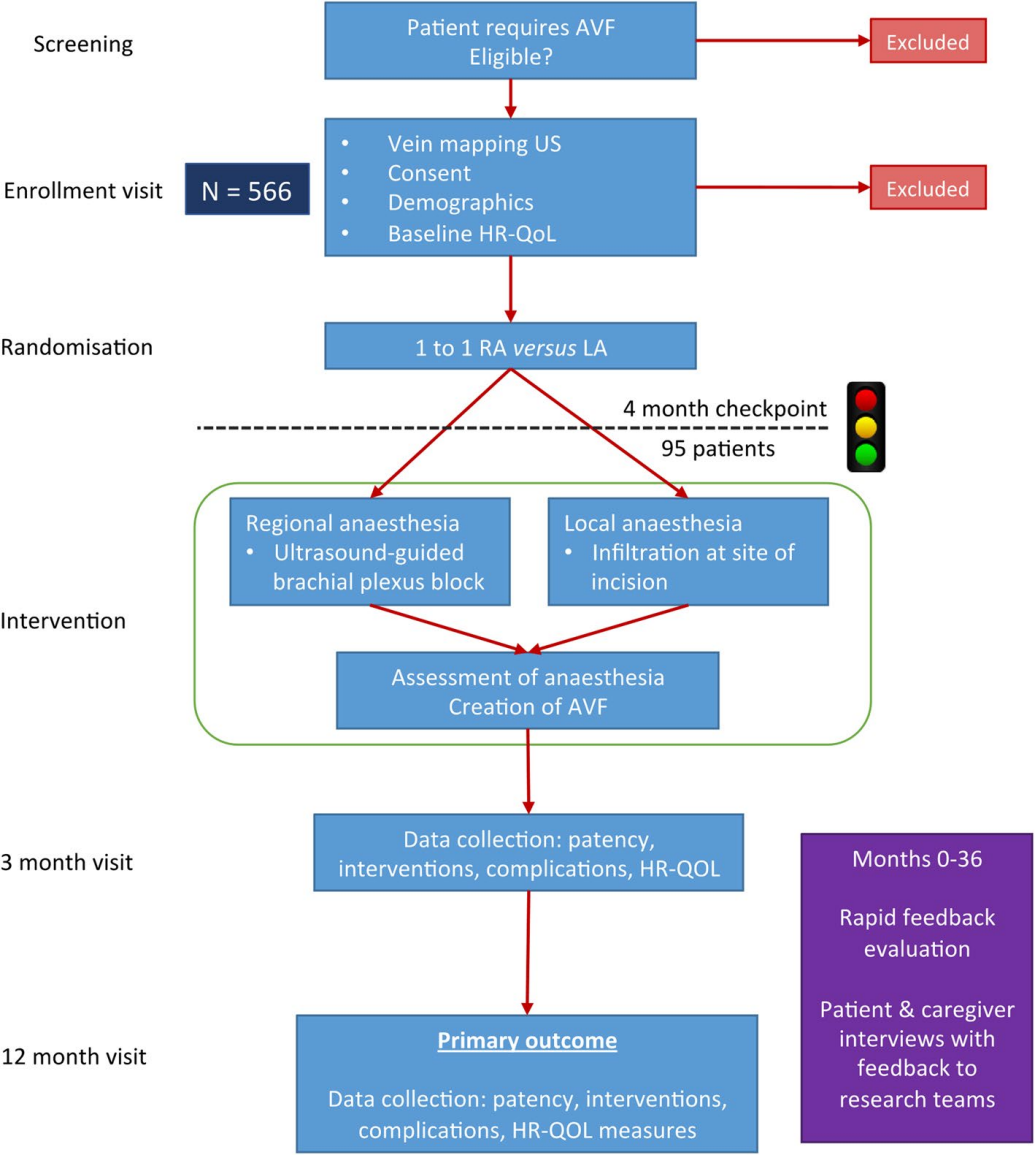
Flow chart summarising the main interventions of the ACCess trial

The ACCess study is a multicentre, single-blinded RCT comparing RA (ultrasound-guided supraclavicular or axillary block) and LA in patients undergoing primary radio- (RCF) or brachio-cephalic (BCF) fistula creation. The primary end point was unassisted functional patency at 1-year (defined as the ability of the access to uninterruptedly deliver the prescribed dialysis without intervention).The study aimed to recruit 566 patients from 20 UK vascular access centres over a 2-year period. Full details of the trial protocol have previously been published (10.1136/bmjopen-2021-052188) [7] and are summarised in the trial flow chart (Fig. 1).

An internal pilot with stop/go checkpoints after 4 months was determined a priori, principally to evaluate the feasibility of recruitment (Table 1).

**Table 1:** Stop–go criteria for the embedded pilot study. After 4 months, the trial was to end if it had recruited < 48 patients, opened fewer than 5 sites or had > 20% failure to trial protocol. If all 12 sites had opened at 12 month and > 95 participants had been recruited, the trial was to continue without the addition of extra sites. We, in fact, achieved the “Amber” criteria at the end of the pilot study, allowing us to expand to include 20 sites. The addition of these extra 8 sites provide hugely valuable in achieving our recruitment targets with nearly 20% of the participants being recruited from the last 8 sites to open

	**Red** Termination of study	**Amber** Continuation with expansion in number of sites from 12 to 20	**Green** Continuation without modification
Number of sites opened	< 5	5–12	12
Total number of participants recruited	< 48	48–95	> 95
Failure of adherence to trial protocol	> 20%	5–20%	< 5%

### Recruitment figures

Recruitment figures were captured monthly by site as the trial progressed. Throughout trial set-up, there was close liaison between the trial team at the co-ordinating centre and lead investigators at each site. Site-specific challenges to clinical service provision in relation to pandemic recovery were recorded monthly and used to prioritise site set-up. The Scottish Haemodialysis Vascular Access Appraisal Report Service Evaluation Tool [15] was used as a measure of the “resilience” of the local vascular access service.

### Embedded process evaluation study

An embedded process evaluation study ran in parallel to the main ACCess trial, utilising a rapid feedback evaluation approach developed by the Rapid Research Evaluation and Appraisal Lab (R-REAL) at the Department of Targeted Intervention, University College, London [16]. This approach has been developed to collect, analyse and share findings with trialists at a time within trial, such that they can be used to inform within trial decision-making processes [17]. The rapid feedback evaluation approach combines qualitative data obtained from semi-structured interviews with patients, carers and staff and documentary analysis (reports, meeting minutes, etc.)

### Data collection

Separate written consent was obtained in person for participants within the process evaluation study. This consent was obtained by a member of the primary research team, such that the first meeting with the interviewer was on the day of the interview.

Semi-structured interviews with patients and healthcare professionals (HCPs) were conducted via secure teleconferencing facilities by a single male researcher from the R-REAL (N.H.). Interviews lasted between 30 and 60 min each. Only the interviewer and interviewee were present at time of the interview. The researcher had extensive experiencing in conducting semi-structured interviews with research subjects but little prior knowledge of the trial specifics or subject matter. Data obtained from interviews were supplemented with documentary analysis (reports, meeting minutes, etc.) and field notes. The team at R-REAL were entirely independent of the main research team responsible for the clinical trial.

Interviews with HCPs explored their experience of setting-up or implementing their trial (depending in their role); the main barriers encountered and strategies implemented to overcome them. Interviews with patients participating in the trial focussed on their experiences of trial participation; understanding of trial literature; experience of treatment options; reasons why they decided to take part in a trial; and (when relevant) reasons for withdrawal. We also sought to interview patients who declined to participate in the main trial; however this was not possible as all approached also declined to participate in the qualitative study.

### Data analysis

The semi-structured interviews were carried out using an interview topic guide. Interviews were recorded, transcribed and imported into NVivo (version 14, Lumivero). Analysis was conducted by two researchers (N.H., C.V.) using framework analysis and findings were summarised using R-REAL sheets [18, 19]. There was no participant checking of data. Interim findings were shared with the Chief Investigator (CI) and Trial Steering Committee (TSC) on three occasions within trial (upon completion of the embedded pilot study; 1-year after recruitment commenced and at completion of recruitment).

### Sample size and sampling technique

A purposive sample of 30–40 staff members (varying professions and specialities (vascular access nurses, surgeons, anaesthetists, research nurses, nephrologists); research roles and a mixture of clinical and academic staff) and 30–40 patients (gender, age, dialysis status, treatment arm) was originally intended however data saturation was obtained with fewer participants and trial recruitment was progressing well, therefore recruitment into the qualitative study was halted after just 15 participant interviews across 5 of the participating centres.

### Approvals and trial registration

Favourable ethical opinion for this project was granted by the West of Scotland Research Ethics Committee Number 3 (20/WS/0178). Both the ACCess study and process evaluation study are registered with the respective clinical trials databases: ISRCTN14153938 and SWAT 150 (MRC SWAT Repository).

## Results

### Recruitment rate

**Fig. 2:**
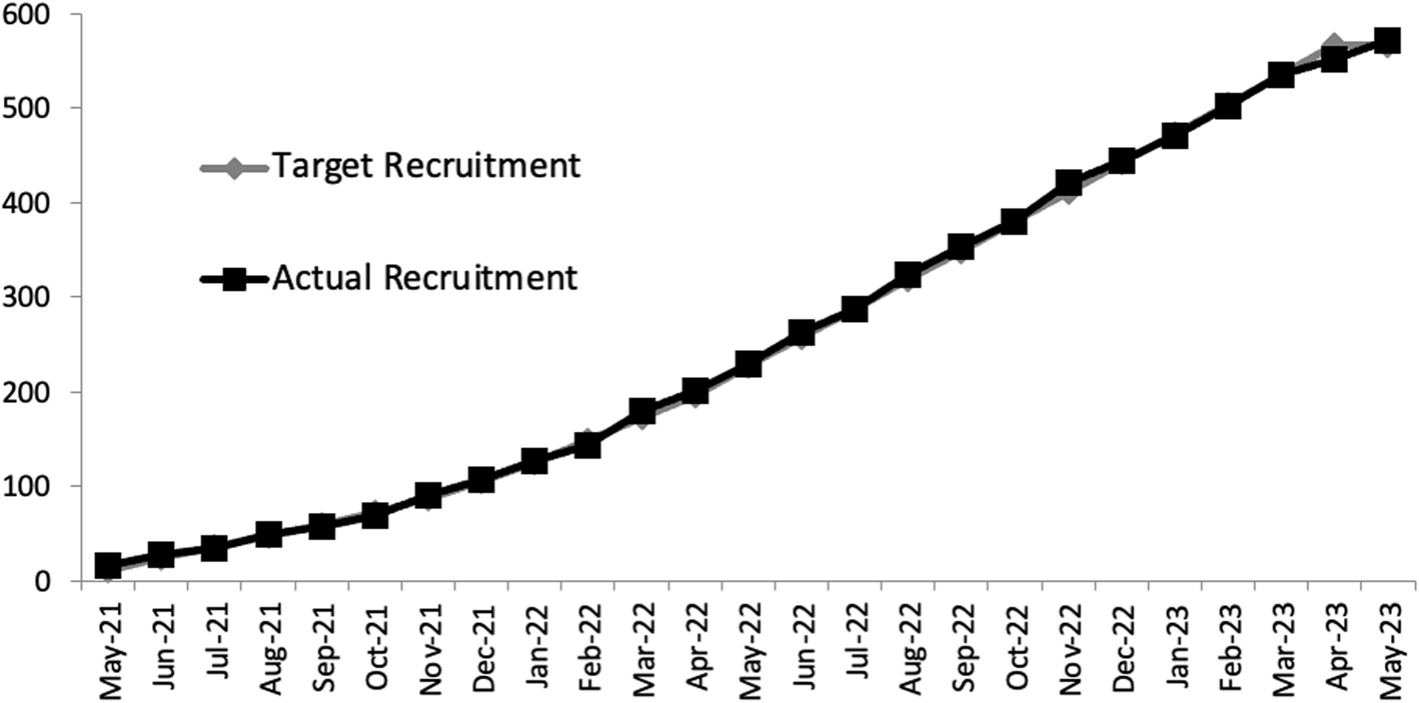
Anticipated (grey) vs actual (black) recruitment by month over the duration of the trial demonstrating that predicted vs actual recruitment was nearly linear and almost exactly the same

**Fig. 3:**
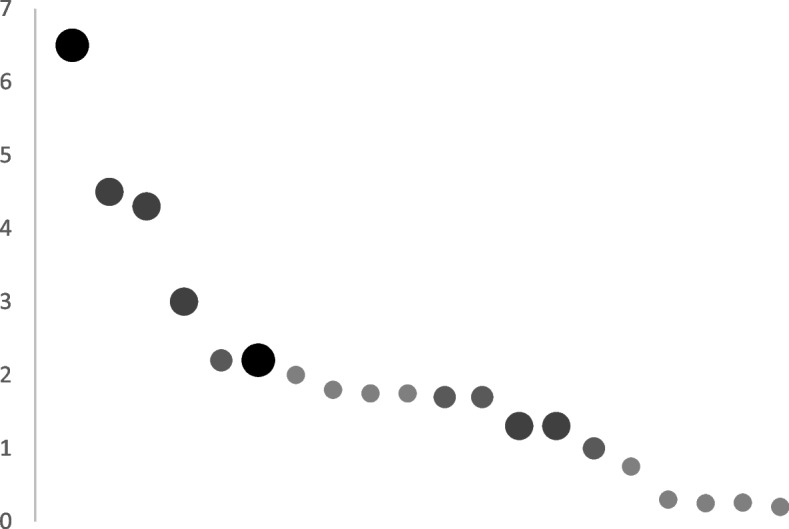
Scatter plot of recruitment rate/month by centre highlighting that, in the main, high volume operative sites were also high volume recruiting sites. The icon denotes centre volume: large black circle which performs > 200 arteriovenous fistulae annually; moderately large dark grey circle denotes a centre which performs 100–200 arteriovenous fistulae surgeries per year; medium sized mid grey circle which performs 50–100 arteriovenous fistula surgeries annually; small pale grey circle denotes a centre which performs < 50 arteriovenous fistulae surgeries annually^a^

The trial closed to recruitment in early May 2023 (24 months after it opened) having recruited 571 patients (101% of target) (Fig. 2). The average rate of recruitment was 2 patients/centre/month, however this varied significantly between centre (range: 0.3–6.5 patients/centre/month) with nearly a third of patients recruited from the co-ordinating centre. The main determinant of recruitment rate was centre volume (Fig. 3). The relationship between centre volume and recruitment rate per month was moderately strong (*R* = 0.65).

Six hundred seventy-eight patients were approached for trial participation, with 116 not participating in the RCT. The most common reasons for patient decline were inability to cope with additional uncertainty/unwilling to be randomised (32%); unwillingness to participate in any form of research (29%); patient preference for one or other anaesthetic technique (28%); and burden of travel and trial visits (10%).

### Site set-up and the embedded pilot study

**Fig. 4:**
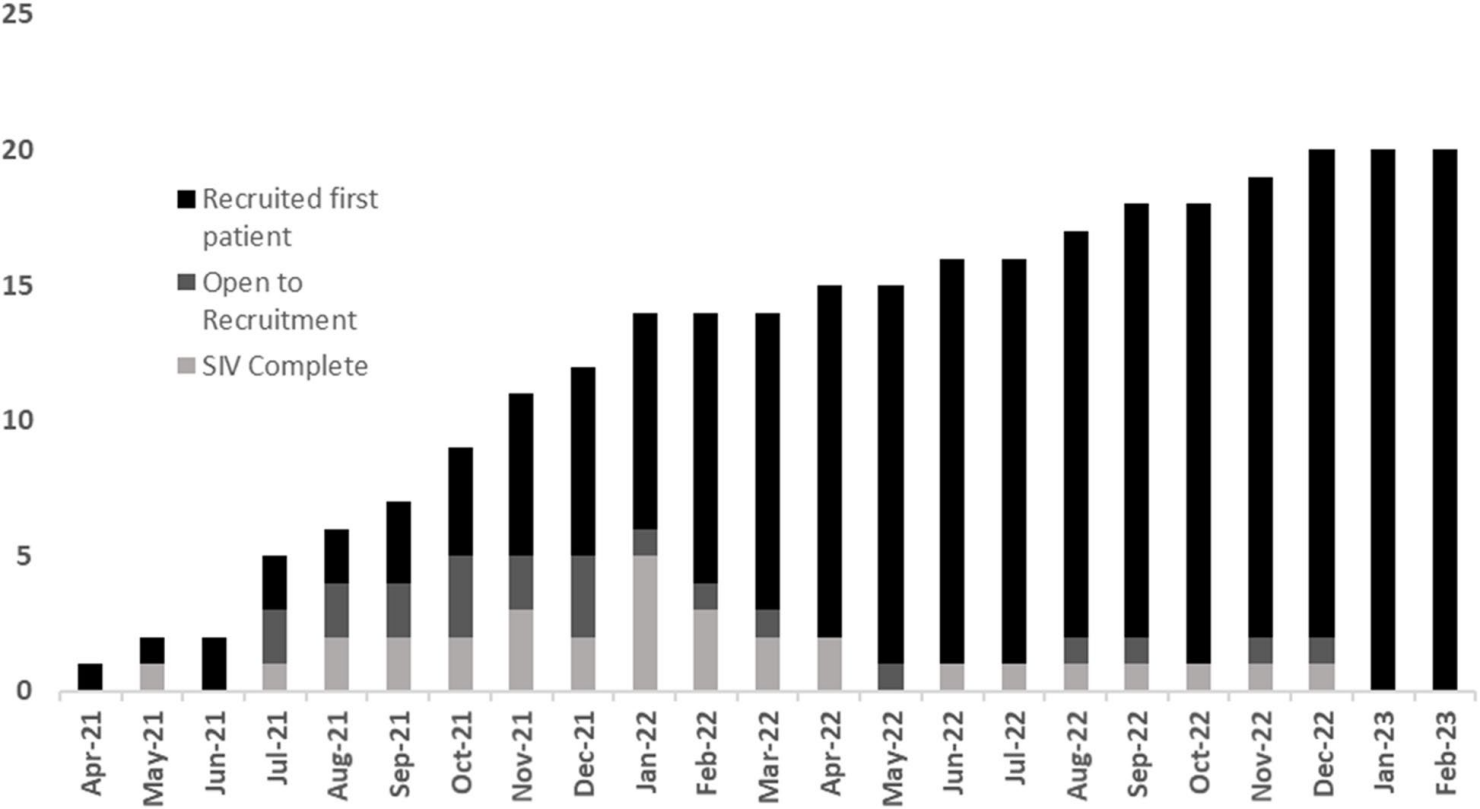
Site-set up by month indicating the number of sites which had undergone a site initiation visit (SIV), had opened to recruitment, and had recruited their first patient by month. This highlights significant delays between site initiation visit and actual recruitment of first patient. Research processes must be streamlined to minimise these where possible. However researchers must also be aware of these and factor them in to deriving achievable recruitment targets

The embedded pilot ran for the first 4 months of the study with stop–go checkpoints as outlined in Table 1. In the early phases of the study, site set-up was slow and there was purposive targeting of Scottish sites, many of which were smaller, as Scotland had been less adversely affected by initial waves of the pandemic [20]. By the end of the embedded pilot phase, there were 5 sites open to recruitment; 49 patients recruited and 100% adherence to protocol. The trial progressed with a decision to expand the number of participating centres from 12 to 20. Priority was given to opening larger centres over the subsequent few months. Thereafter, sites opened at a fairly consistent rate (Fig. 4).

### Process evaluation study

Twenty-three patients (13 who participated in the study and 10 who declined) and 12 HCPs were approached to participate in the process evaluation study. Four patients and 11 HCPs consented to participate. All patients interviewed had either completed the trial or were still participating at time of interview. Only one patient who declined participation in the main trial initially agreed to participate in the qualitative study, but subsequently changed their mind prior to interview.

The main themes identified supporting trial participation were as follows: a desire to improve patient care; clarity of communication; strong leadership and simplicity of trial design. Tables 2 and 3 outline the main motivators for trial participation and enablers of trial processes.

**Table 2:** Factors motivating patient and HCPs desire to participate in the ACCess study and supporting quotations from patients/HCPs

***Patient*** Desire to participate in research Clear, simple communication Trust/attitude of HCPs	Research participation viewed as a way to improve care “needed to improve care in the future” (Patients 1,2,3) “I feel I can give something back” (Patient 4) Clear trial documentation Good verbal discussions Appropriate trial acronym “extremely well explained in every way” (Patient 3) “acatchy name helps you remember” Longstanding clinician-patient relationships Importance of trust in the first approach “It’s nice to have someone you know and trust (Patient 1) “Positivity of staff (making the first approach) important” (Patient 2) “I didn’t really read the information of think about the consent process particularly. I trusted Dr. X” (Patient 2)
***HCPs*** Desire to improve patient care Desire to participate in research Important question	Ownership of patient care very evident Research participation viewed as a means to improve care “For our patients”/“In our area” (all HCPs) “nice to be doing something interesting in (their own speciality) again, rather than just doing pandemic stuff” (HCP 1) “Research is essential for healthcare to advance” (HCP 5) Importance of outputs for career progression Value of Associate PI scheme “I mean, people look at these things when you’re looking for jobs in the future. I think it’s helpful to have something to show for your work.” (HCP 6) All researchers considered it to be an important question Most considered equipoise, though one made reference to our previous single centre RCT and considered that this may have already changed practice such that a further trial was not needed “Personally, I think we already have enough evidence. I’ve changed my practice already.” (HCP 1)

**Table 3:** Barriers and enablers to delivery of the ACCess study and supporting quotations from patients and HCPs

Enablers	Simplicity Flexibility/pragmatic trial design Communication Leadership Engagement	Simple trial protocol with little deviation from standard care High volume procedure Clear study documentation “well designed, well presented protocol” (HCP 2) “The study documents were very clear” (HCP 5) “The PIS provided a useful aide memoir when going through the consent process” (HCP 10) Pragmatic trial design The protocol was flexible to accommodate local pathways and processes of care Trial visits minimised and co-ordinated with existing hospital visits/dialysis sessions if possible Adoption of remote consent following within trial feedback Digital study documents felt may be beneficial in the future “efficient trial processes could easily be incorporated into our routine clinical care” (HCP 1) “there were definitely enough patients coming through the clinics to meet the recruitment targets” (HCP 3) “Because of the COVID backlog in cases, we have loads of patients that we have already seen in clinic, know are eligible to participate in the trial, but whom, we don’t want to have to bring back to the hospital to discuss the trial with them. It would be great to be able to consent them remotely” (HCP 4) “We don’t have anaesthetists available for every theatre list. If we could consent patients (remotely), in advance, it’d let us randomise them and assign them to the best theatre list in advance and save a lot of resource” (HCP 5) “Digital information about the trial might be helpful for patients that perhaps have reading or eyesight difficulties” (Patient 3) Open channels of communication between the co-ordinating team and sites permitted a better understanding of site specific challenges. This facilitated prioritisation of site set-up and help trouble shooting problems Regular trial meetings and “drop in” sessions were established following within trial feedback. Research nurses found this particularly helpful to troubleshoot problems with data entry and facilitate shared learning between sites “The drive and support from the central team was essential” (HCP 2) “More regular communication with the central team and other sites would be helpful just to discuss trial progress, keep everyone in the loop and have a point of contact if problems arise” (HCP 3) Good communication and leadership from the co-ordinating centre Engaged and driven Chief Investigator “strong leadership of the Chief Investigator” (HCP 4) “Drive and communication from the central team” (HCP 4) “The Chief Investigator and Project Manager are very approachable.” (HCP 3) “The enthusiasm of the team in Glasgow really drives things forward” (HCP 3) Having the right people involved was essential with engagement from every part of the multidisciplinary team from the outset “A Research Nurse joining the team was pivotal to take the burden off the vascular surgeons” (HCP 4)
Barriers	Research burden Limited resources Research bureaucracy Engagement	The renal patient cohort is unique in teams of underlying treatment burden. HCPs expressed concerns that additional scans, hospital visits etc. might put patients off participation QOL questionnaires considered especially burdensome It was suggested that electronic documentation may help reduce this burden As the trial was unblended, patient’s may be unhappy with the arm that they were allocated to” “Our patients are already coming to dialysis three times a week. They don’t want any extra hospital visits or scans. Especially with the pandemic, many are anxious about coming to the hospital at all.” (HCP 1) “The patients hate filling out the QOL questionnaires, especially the KD-QOL. They are too long and complicated for our patients who are often frail and don’t want to spend a lot of time going through paperwork. We’ve had a lot of unanswered questionnaires returned” (HCP 5) “Some of our patients have a preference about which anaesthetic they want. We’ve had one or two patients withdraw from the trial after randomisation because they didn’t get randomised to the anaesthetic (technique) that they obviously wanted” (HCP 2) Many sites were adversely affected by the pandemic (Table 4) Staffing turnover and redeployment particularly of nursing staff was a challenge Limited availability of ultrasound slots Variableaccess to theatre lists with availability of an anaesthetist to provide RA/recovery areas “There was limited capacity of scans in the lab” (HCP 3) “We suffered from the wider pressures on the NHS- COVID related and acute staffing issues” (HCP 2) “We had limited access to theatres and recovery areas due to changes brought about by the pandemic” (HCP 1) “Areas of the hospital were being rebuilt and were under construction meaning that we had limited access to recovery areas and limited anaesthetic capacity to do the blocks” (HCP 6)” Credit for accruals can currently only be allocated to one speciality area and not shared between specialities; this can lead to internal frictions and is counterproductive to studies, like ACCess, that require multidisciplinary collaboration Failure to prioritise research in pandemic recovery plans “Research was not prioritised in the pandemic recovery plan” (HCP 4) Early engagement of all relevant personal is particularly important when delivering multidisciplinary studies; implementation of this was variable between sites Added value of research nurses Need for strong leadership and well-functioning team to promote good recruitment. Concerns expressed that at sites where this infrastructure was not so well established that recruitment might prove harder The definition of “block failure” may dissuade participation from anaesthetists Positive attitude of co-operation and collaboration between centres (viewed as a UK-vascular access community project) and between specialities within centres was necessary (this was viewed as a driver in some cases and a barrier in others) “We had variable support from the anaesthetists” (HCP 3) “I’m not sure if recruitment will be as good at other sites when the CI isn’t there to drive recruitment” (HCP 1) “There may be less buy-in from the surgeons (or the anaesthetists, or the nurses) at sites that don’t have such a well-established vascular access team as we do” (HCP 1) “Different units have different relationships within their vascular access MDT” (HCP 5) “It’s the definition “block failure”. I think it puts anaesethetists off or might cause them to alter their practice to avoid being labelled a “failure”. I don’t think the term “failure” in the protocol was helpful.” (HCP 2)

Patients were generally positive about their experience of participation. The main motivator for participation in the study, for both patients and HCPs, was to “improve care” for patients with kidney disease (Table 2). Patients acknowledged that the chronicity of their renal failure, meant that developments in kidney care now may have a personal impact in the future. Others were motivated by a desire to “give something back” to a healthcare system that they perceived to have served them well.

Strong relationships between patients with renal failure and HCPs were apparent. All of the HCPs interviewed expressed some form of ownership for patients and their outcomes e.g. “for our patients”/“in our area”. Trust in the person making the initial approach for consent was considered an important factor in the decision whether or not to participate. Notably in the four highest recruiting centres, it was the clinician responsible for the clinical care (rather than a dedicated member of the research team) who made the initial approach. Conversely, one HCP considered it “essential to have a research nurse to take the stress off the surgeons”.

Patients considered that “positivity of staff” making the initial approach, along with simplicity of explanation and trial literature were important factors influencing their decision to participate with one participant describing the trial as “extremely well explained in every way”. Another found that a “catchy name (acronym) helps you remember”. HCPs reported that good communication, strong leadership and a pragmatic trial protocol were among the most important enablers of trial delivery. From early in the trial design phase, it was recognised that the potential pool of participants was huge, given AVF creation is such a high volume procedure. The challenge was in enabling recruitment within a busy clinical service. Two-way communication between co-ordinating centre and site teams within the design and set-up phase of the trial and pre-existing knowledge of the clinical pathways within the various participating centres, facilitated a pragmatic trial design that, whilst ensuring standardisation of the trial intervention, allowed for local variation in consent procedures and timing of pre-operative work-up etc. so that trial processes align with existing clinical pathways and avoid unnecessary deviation from standard practice. These efforts were reflected in feedback within the process evaluation study with one HCPs reflecting that “efficient trial processes could easily be incorporated into our routine clinical care”. Regular trial newsletters and social media updates were utilised to maintain engagement with research teams as the trial progressed with the importance of an engaged and visible Chief Investigator highlighted by several HCPs as important to the success of the trial. Notably however, others felt that at sites where the CI wasn’t present, lack of this driving force maybe felt. As a result of this within trial feedback we established online “drop-in sessions” (giving researchers at participating centres the opportunity to have regular contact with the CI; share experiences and learning; and troubleshoot problems as they arose).

Potential barriers to effective trial delivery were identified as follows: the burden of research visits on participants (particularly in the case of haemodialysis patients already attending for dialysis three times per week); inadequate resources; research bureaucracy; and inadequate engagement of all the necessary research personnel/team members (Table 3).

Our Patient and Public Involvement and Engagement (PPIE) in the design phase of the project had prior identified the burden that additional trial visits might pose on a dialysis population already attending the hospital for 4 h three times per week for maintenance haemodialysis. This had informed a protocol that minimised the need for extra hospital visits and encouraged follow-up within the dialysis unit, during dialysis sessions, wherever possible. Despite this, patients who had not yet started dialysis still required additional hospital attendances for scans which may have disincentivised participation. HCPs also noted that the specific commitments of the trial, in particular completing quality of life questionnaires, became too tiresome for many of the older, frailer patients. Patients recommended the option of electronic study documents for future similar studies and noted that this would be particularly useful for patients with comorbidities (e.g. visual or writing impairment).

In response to within trial feedback from the process evaluation work (Table 3) and supported by the positive experiences of the COVID-19 trials, remote consent was implemented at the mid-point of the recruitment phase. In total, 136 patients were recruited by remote consent. Remote consent proved helpful to minimise additional hospital visits (in particular at sites where a separate research team was obtaining consent) and provided an additional pool of eligible patients whose surgery had been delayed as a result of the pandemic to be approached for participation without necessitating another clinic appointment. At sites which had previously been obtaining consent on the day of surgery, it also permitted earlier randomisation and streamlining of theatre lists as it was possible to determine which patients needed an anaesthetist to perform the block in advance.

Staffing shortages, lack of access to theatre, limited capacity for imaging and variable anaesthetic support were all identified as potential barriers to effective trial delivery. Many of these were variably observed across sites and reflected wider pressures on the NHS following the COVID-19 pandemic (Table 4).

**Table 4:**
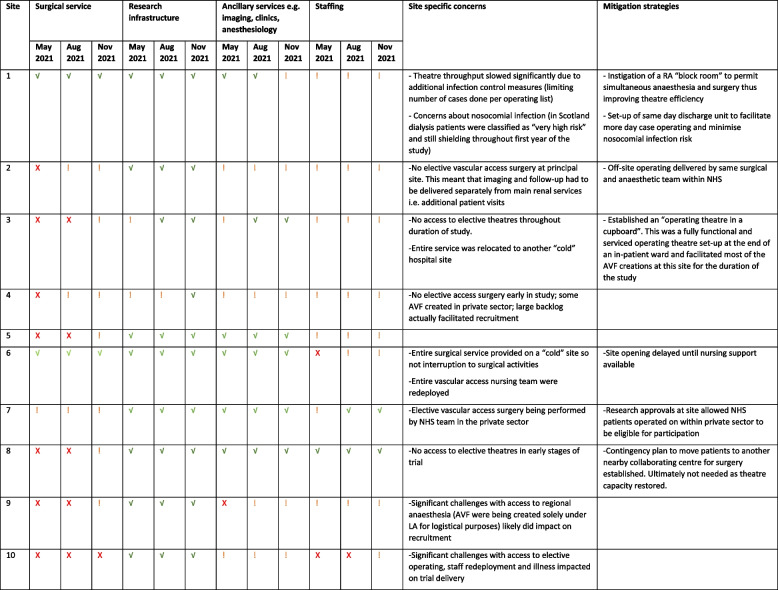
Site-specific challenges during site set-up and recruitment and example mitigation strategies employedb. The table outlines availability of elective operative capacity; local research infrastructure to set-up and deliver non-COVID research at the time; availability of ancillary services need to deliver the trial (imaging, regional anaesthesia etc); staffing pressures at three different time points during the first year of the trial. A green tick (  ) indicates full service with no logistical issues; an amber exclamation mark (  ) indicates some pressures on the service; and a red cross (  ) indicates that there was no access to this aspect of the service at that time. Knowledge of these factors was utilised to prioritise site set-up

Additionally, we had utilised the term “block failure” within the protocol as a simple way to define a block that did not achieve the desired level of anaesthesia. Several HCPs interviewed felt that the terminology “failure” could be regarded negatively with anaesthetists feeling as if their practice was being scrutinised or audited. This could result in poor engagement or alteration of practice in order to avoid the perceived “failure”*.*

### Impact of the COVID-19 pandemic, site “capacity” and local “site specific” barriers

Throughout trial set-up, the co-ordinating team liaised closely with sites in order to understand local situations, address potential barriers and prioritise set-up of sites that were in a position to deliver the trial effectively at that time point. This was particularly relevant given that the Delta-wave of the COVID-19 pandemic hit the UK between May–September 2021 [21].

Each site was unique in terms of the challenges it faced in set-up and delivery of the trial. These challenges evolved with time, such that the same site often faced different barriers to trial delivery at different time points (Table 4). Some of these barriers were pandemic-specific (e.g. restrictions placed on non-COVID research; cessation of elective surgery, etc.). Some challenges originated from the pre-existing infrastructure to deliver the clinical vascular access service (in some cases exacerbated by the pandemic) e.g. capacity to deliver imaging; multidisciplinary working etc. Sites with the most complete clinical infrastructure prior to the pandemic, proved most “resilient” to the additional pandemic-specific challenges in relation to trial delivery (Tables 4 and 5).

**Table 5:** Recruitment rates and vascular access appraisal report service evaluation tool scores (total maximum score shown in brackets) broken down by domain for each participating site^b^

**Site**	**Recruitment rate** (patients/centre/month)	**Vascular access service evaluation tool domain**						**Overall vascular access service evaluation tool score (63)**
		**Governance (7)**	**Job planning (2)**	**Service provision (13)**	**Education (7)**	**Patient experience (2)**	**Processes (32)**	
1	6.4	3	1	7	6	2	21	40
2	4.5	0	2	4	4	0	10	20
3	4.4	6	1	8	7	2	20	44
4	2.3	4	0	5	6	1	13	29
5	1.7	3	1	7	6	2	17	36
6	1.5	5	0	2	4	1	6	18
7	1.2	7	2	4	5	1	16	35
8	0.7	5	0	6	6	2	17	37
9	0.3	2	0	3	5	1	7	18
10	0.3	2	0	2	5	1	14	24

We observed some excellent examples of innovation e.g. establishment of an anaesthetic “block room” to improve theatre efficiency; and rapid establishment of research agreements to facilitate trial participation of NHS patients having surgery within the private sector. In the absence of access to operating theatres, one site even converted a cupboard at the end of their in-patient ward into a vascular access operating theatre. At least in part, desire to participate in the trial served as a catalyst to deliver these improvements in clinical service provision. Similarly, a number of anaesthetic and theatre nursing staff (many of whom had been redeployed early in the pandemic) found that the trial provided a morale boast and that it was “nice to be doing something interesting in (their own speciality) again, rather than just doing pandemic stuff”.

Ten sites responded to the request to complete a Vascular Access Appraisal Report Service Evaluation Tool [15]. There was no association between overall Vascular Access Service Evaluation Tool score and recruitment rate (*R* = 0.35). However certain questions, primarily those within the “Process” domain of the tool, did appear to be associated with both a site’s ability to recruit patients and speed of recovery of the clinical service (*R* = 0.5). All sites with a recruitment rate of > 2 patients/centre/month had a named vascular access lead for nephrology, surgery and interventional radiology; regular multidisciplinary team (MDT) meetings; formal processes to audit vascular access outcomes and co-location of nephrology, vascular access surgery and interventional radiology services on the same site (Table 5).

## Discussion

Despite the challenges of delivering a complex perioperative intervention within the post-pandemic NHS operating theatre environment, the ACCess study has recruited on time and on target. It is the largest RCT of vascular access in Europe to date. We have demonstrated that there is a significant will within the UK vascular access community to deliver pragmatic clinical trials aimed to directly improve patient care. Although the exact volume of vascular access surgery in the UK is unknown, it is likely that 15,000–20,000 simple AVF are created annually (G. Pettigrew, personal communication), making it one of the most commonly performed vascular procedures and ensuring that there are many potentially eligible patients for this, and similar, studies. Murphy and colleagues (2021) have demonstrated that a will and desire to participate in clinical trials (even those of complex interventions) exists among patients with advanced renal disease [22]. Despite this, historical recruitment into clinical trials of vascular access has been poor with many trials terminated early. Only 27 of the 50 most recent clinical trials of vascular access listed on the National Clinical Trials database (ClincalTrials.gov) completed recruitment. The major impediments for trials of vascular access seem to be perceived lack of equipoise by clinicians; difficulties standardising interventions and/or operative technique across multiple sites; and overly stringent inclusion criteria [5, 23]. How has the ACCess study managed to overcome these perceived barriers where others have failed?

**Fig. 5:**
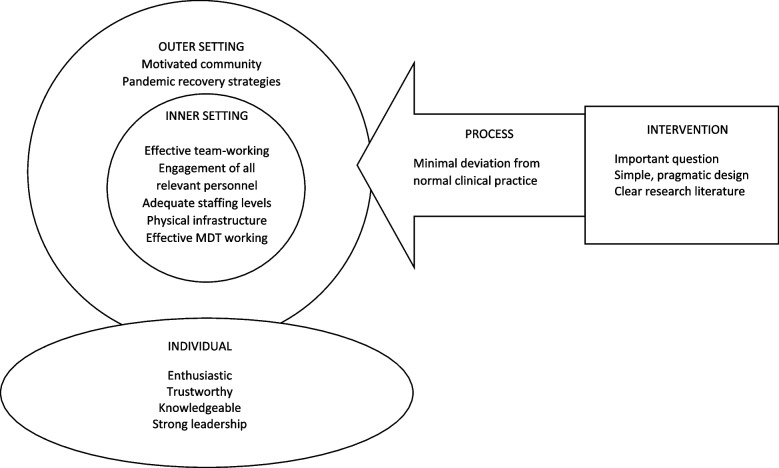
A summary of the main facilitators of successful implementation of the ACCess study utilising the five CFIR domains [26]

Implementation science is a novel area of research which seeks to understand why an intervention (is this case a research study) succeeds or fails [24]. Many interventions fail despite well-developed plans for execution because contextual factors can be powerful forces acting against real-world implementation [25]. Clinical trials serve as dynamic, complex contexts in which the reasons for success or failure are often multifactorial. The Consolidated Framework for Implementation Research (CFIR) can be utilised to help researchers understand and address the facilitators to implementation [26]. Figure 5 outlines the enablers within the ACCess study in line with CFIR framework and domains.

The ACCess study took a pragmatic approach to study design and tried to deviate as little as possible from routine care. This both made it easier for clinicians to incorporate research into clinical practice and will ensure results are translatable “real-world” clinical practice. For example, whilst the anaesthetic and surgical technique were mandated to ensure validity and reproducibility, the protocol was not prescriptive with regards to timings of perioperative interventions (e.g. pre-operative ultrasound etc.), thus allowing individual sites to deliver the trial within their existing local clinical pathways in the most part. This allowed many sites to deliver the trial utilising entirely clinical staff, whilst others found the addition of dedicated research nurses pivotal to successful recruitment.

A complex interplay exists between the delivery of clinical research and the provision of clinical care. We previously observed this in our RCT comparing lines and grafts for dialysis [27] (a trial that could not have been delivered without a rapid expansion of our clinical graft practice; however that expansion would not have occurred at the speed it did were it not for support and desire to participate in the trial). The ACCess study also provides many interesting examples of this synergy. A number of sites observed that the trial served as a catalyst for cross-speciality collaboration (between surgical and anaesthetic teams) in the post-pandemic recovery period. Several sites reported that a desire to deliver the trial served as a driver for innovation in the post-pandemic recovery period, the results of which actually served to improve patient care. For example, several sites developed “block rooms” which increase theatre throughput and one site created a makeshift “operating theatre in a cupboard”, which facilitated surgeries that would otherwise have been delayed.

The ACCess study has highlighted the massive variation in vascular access service provision around the UK. Vascular access care is a complex multidisciplinary process which, as data from the trial highlights, is only as good as the weakest link in those processes. Different units faced different challenges, at different time points (Table 4). However, implementation science and the CFIR model [26] (Fig. 5), necessitate that the ability to deliver both the trial and clinical care relies on all parts of the service functioning simultaneously. The existence of a cohesive MDT (in particular the conduct of regular MDT meetings) appeared to be a surrogate for a site’s ability to implement all parts of the CFIR model and thereby to deliver the trial effectively. This may be a unique feature of the ACCess study, given its multidisciplinary nature. However it’s more likely that the infrastructure needed to deliver efficient research is similar to that needed to deliver a clinical service. If so, service evaluation tools may prove useful measures of research capacity in future trial planning.

The rapid review and evaluation approach adopted within the process evaluation study permitted timely evidence synthesis and feedback to and inform “within trial” changes and modifications [18, 19]. In response to feedback, we were able to implement “remote consent” at the midway point of the trial, which facilitated recruitment of nearly one third of patients and adopt videoconferencing “drop-in” sessions which permitted timely troubleshooting of problems, made the central trial team more accessible and promoted shared learning between sites. Additionally, an awareness that some HCPs perceived negative connotations of the term “block failure” allowed us to address this within later site set-up visits (offering assurances that an individuals practice was not being audited).

The vast majority of patients recruited had CKD-V and were deemed within 6–12 months of needing to commence HD. A much smaller proportion had already started dialysis at the time of recruitment. Complex multimorbidity is common in this patient group with nearly 60% of patients with CKD-V having four or more long-term conditions [28]. This gives patients with advanced kidney disease a unique treatment burden associated with out-patient clinic attendances and unplanned hospitalisations, even prior to commencing three times weekly hospital attendances for HD. Transitions between dialysis states are negatively associated with health-related quality of life (HR-QOL) [29] and there is considerable anxiety and uncertainty associated with the time period around starting dialysis [10, 11]. This is reflected in the reasons given for trial non-participation, with nearly a third declining because they could not cope with the additional uncertainty associated with randomisation (preferring that the clinician chose “the best option” for them). Anecdotally, some patients found the concept of equipoise distressing. Considerable efforts were made within the ACCess study protocol to minimise the research burden for participants: once commenced on HD follow-up visits could be co-ordinated with dialysis sessions; there were no blood tests and only minimal scans etc. Despite this, many patients found HR-QOL questionnaires too burdensome. This is an important consideration for the selection of tools to measure patient related outcome/experience (PROMS/PREMs) in future clinical trials within this patient cohort. Interestingly, despite advancing age and frailty of many participants, the process evaluation study indicated that there may actually be a reasonable level of IT literacy within this population, making electronic documentation and/or digital follow-up an option for future studies in this patient group.

## Conclusions

In conclusion, despite anticipated challenges, the ACCess study has recruited on time and on target. In-depth knowledge of the patient group gleaned from prior research in this area; good communication; early engagement of a broad multidisciplinary team and pragmatic protocol mimicking standard clinical care proved fundamental to our success. Implementation science acknowledges that even well-developed plans may fail due to contextual factors and the success or failure of an intervention (clinical trial) necessitates all CFIR domains to function in parallel. Our work suggests that a well-functioning MDT serves as a good surrogate for “resilience” (i.e. all CFIR domains can be delivered). These factors should be considered by researchers considering undertaking future clinical trials of complex interventions.

## Data Availability

The complete dataset from the ACCess study will be available via NIHR Open Data repository following data lock. https://nihr.opendatasoft.com/explore/dataset/infonihr-open-dataset.

## Abbreviations

AVF: Aarteriovenous fistula
BCF: Brachiocephalic fistula
CI: Chief Investigator
CKD-V: Chronic kidney disease stage 5
CRIF: Consolidated Framework for Implementation Research
HCPs: Health care practitioners
HD: Haemodialysis
HR-QOL: Health-related quality of life
LA: Local anaesthesia
MDT: Multidisciplinary team
NHS: National Health Service
NIHR: National Institute of Healthcare Research
PPIE: Patient and Public Involvement and Engagement
PREMs: Patient Related Experience Measures
PROMs: Patient Related Outcome Measures
RA: Regional anaesthesia
RCF: Radiocephalic fistula
RCT: Randomised controlled trial
R-REAL: Rapid-Research Evaluation and Appraisal Lab
RRT: Rrenal replacement therapy
TSC: Trial Steering Committee
UCL: University College London
